# Supplementary material for: Direct printing of functional 3D objects using polymerization-induced phase separation
Source: Nat Commun. 2021 Jan 4;12:55. doi: 10.1038/s41467-020-20256-3 (PMC7782741; doi:10.1038/s41467-020-20256-3)
Supplement: Supplementary file 1 — Supplementary Information [file 41467_2020_20256_MOESM1_ESM.pdf]

Supplementary Information

Direct Printing of Functional 3D Objects Using Polymerization-Induced Phase Separation

Bhavana Deore,[1]* *Kathleen L. Sampson,*[1] *Thomas Lacelle,*[1] *Nathan Kredentser,*[1] *Jacques Lefebvre,*[1] *Luke Steven Young,*[1] *Joseph Hyland,*[2] *Rony E. Amaya,*[2] *Jamshid Tanha,*[3] *Patrick R. L. Malenfant,*[1] *Hendrick W. de Haan,*[4]* *and Chantal Paquet*[1]*

[1]Security and Disruptive Technologies, National Research Council Canada, 100 Sussex Drive, Ottawa, ON, K1A 0R6 Canada

[2]Department of Electronics, Carleton University, 1125 Colonel by Drive, Ottawa, ON, K1S 5B6, Canada

[3]Human Health Therapeutics Research Centre, National Research Council Canada, 100 Sussex Drive, Ottawa, ON, K1A 0R6 Canada

[4]Faculty of Science, University of Ontario Institute of Technology, Oshawa, ON, L1H 7K4, Canada

*E-mail: Bhavana.Deore@nrc-cnrc.gc.ca; Chantal.Paquet@nrc-cnrc.gc.ca; hendrick.dehaan@ontariotechu.net

List of Tables

Supplementary Table 1. Resin components and their solubility parameters. 4
Supplementary Table 2. Composition of polymerizing components of photoresins. 5
Supplementary Table 3. Resistance as a function of different wt % of silver precursor (AgND, silver neodecanoate and 2-ethyl-2-oxazoline) in 35 wt % crosslinker DA-250 formulations and their sintered samples. 6
Supplementary Table 4. Light intensity and estimated light doses of the laser used for printing and gelation experiments. 21

List of Figures

Supplementary Figure 1. Optical microscope images of cylinder printed using 25, 30 and 38 wt % AgND and using resins containing 35 wt % crosslinker DA-250. 7
Supplementary Figure 2. TGA of 3D printed and sintered cylinders in air. Cylinders are printed (a) without and (b) with 25 wt % of AgND in the resin. 8
Supplementary Figure 3. SEM images, EDS layered electron images and EDS mapping of silver, carbon and oxygen elements of the cross-sections of pre-sintered cylinders made from two different wt % DA-250 resin compositions. Cylinders were dried for two days in fume hood at room temperature. 9
Supplementary Figure 4. a) The Ag wt % as a function of distance away from the surface along the cross-section of a 1.5 mm diameter cylinder. The center of the cylinders made with 25 wt % crosslinkers have as low as 4 wt % Ag while those made with 99% crosslinker have silver concentrations with 15-20 wt % within its core. b) SEM image of the center of 99 wt % DA-575 showing inclusions of silver. 10
Supplementary Figure 5. SEM images of the surface of dipole antenna prepared using (a, b) phase-separated and (c, d) electroless plating method. The images show the higher surface roughness of the electroless-plated sample compared to the phase separated samples. Roughness values found using optical profilometry show the phase separated samples are one order of magnitude lower than that of electroless deposited samples. Optical microscope images of the samples after a tape adhesion test showing the improved adhesion of the silver coating on samples prepared using phase separation (e) over electroless plating (f). 11
Supplementary Figure 6. Delay time of DA-575 resin with and without Ag precursor as a function of wt % of crosslinker measured using the optical microscope setup. We note that experiments performed using photoresins with AgND show similar trends in t_d as a function of wt % of crosslinker compared to those performed without AgND. 12
Supplementary Figure 7. Solubility parameters of monomers, crosslinkers and the silver complex estimated using group contribution methods. 13
Supplementary Figure 8. The elastic modulus of the various polymers using 25 wt % crosslinker. With increasing crosslinker segment, the elastic modulus decreases. Elastic modulus was determined from tensile tests on samples approximately 1.5 mm in diameter and 1 cm in length. The test instrument, a LEX820/FDAS770 automated fiber tensile test system from Dia-Stron (Andover, UK), measured the sample cross-section using an on-board laser micrometer followed by tensile testing using a high resolution extensometer with 20 N load cell. Four samples of each type were measured to report the average result and standard deviation. 14
Supplementary Figure 9. a) Isometric, top and side views of the truss structure used in the strain sensor experiments. b) Photograph of 3D printed truss with dimensions of 11.24 x 11.24 x 13.40 mm. c) Relative

resistance of trusses under compression for trusses made with resins containing various crosslinking concentrations. 15
Supplementary Figure 10. (a) Gain standard horn antenna (BAE Systems H-1498). (b) Measured antenna array gain compared with HFSS simulation. (c) HFSS model of dipole antenna array with feeding network and metal conductivity of 1×10^6 Siemens/m. 17
Supplementary Figure 11. An SEM image of the surface of 3D printed the anti-microbial sample made from resins containing a) 0.5 wt % and b) 1.0 wt % AgND. 18
Supplementary Figure 12. (a) Photographs of the bacterial inhibition zones created by the different scaffolds against TG1 (E.coli) and (b, c) bacterial growth kinetics in liquid medium of E.coli. 19
Supplementary Figure 13. (a) Variation of the titanium as a function of distance from the surface of a cylinder printed with TiO_2 nanoparticles and as measured by EDS. (b) SEM image of surface of cylinder printed with TiO_2 nanoparticles and image of printed cylinders (insert). (c) Cross-sectional SEM image of a cylinder printed with TiO_2 nanoparticles. (d) Cross-sectional SEM image of a cylinder printed with barium strontium titanate nanoparticles. (e – f) SEM/EDS cross-section image of a cylinder printed with iron oxide nanoparticles. The nanoparticles appear as bright areas in the SEM. EDS mapping of carbon (f) and iron (g) elements in the sample. 20
Supplementary Figure 14. Resistance of cylinders printed using different light intensities for DA-250 resins. 22
Supplementary Figure 15. TGA of pre-sintered 3D printed cylinders using a resin mixture (75 mL of 50 wt % DA-575 with 25 mL of 35 wt % DA-250) that contained no silver and treated with 5 minutes of UV curing. The mass loss of the cylinders were analyzed by TGA under isothermal condition at 210°C (i.e. sintering temperature). The mass loss after 1 h is 1.4% demonstrating that the polymer does not degrade significantly during the sintering step. 23
Supplementary Figure 16. Optical microscope setup for examining the formation of the polymer network and measuring delay time upon exposure to a 405 nm laser. 24
Supplementary Figure 17. Images of the initial state of the system for crosslinkers of length 3 (left) and length 9 (right). The probe molecule is shown in green and is represented as it is modeled: three beads connected by springs. For clarity, the crosslinker molecules are shown as tubes such that individual molecules are easier to distinguish. Note however that these molecules are also modeled as beads connected by springs. These images are cross-sections selected to show the location of the probe molecule. Instances of shorter crosslinker molecules, or even single beads, are an artefact of cross-sectional view of molecules. 25
Supplementary Figure 18. Experimental setup for the strain sensor measurement. The sample dimensions are 11.24 x 11.24 x 13.40 mm. 26

Supplementary Note 1. Materials and Resins

The monomer and crosslinkers were purchased from Sigma Aldrich (Oakville, ON, Canada). The concentration of inhibitor in the crosslinkers ranged from 0 to 0.006 wt % while the monomer, EHA, contained 0.004 to 0.11 wt % inhibitor. The inhibitor was left in the resins in order to ensure greater consistency from print to print. A molar concentration of photoinitiator of at least 6 times that of the inhibitor was used to ensure the inhibitor did not have a significant effect on polymerization rates. The photoinitiator, ethyl (2,4,6-trimethylbenzoyl) phenylphosphinate (TPO-L), and 2-ethyl-2-oxazoline were purchased from Sigma Aldrich. The silver neodecanoate was prepared as described in the literature.[1] The structures, names, molecular weights and solubility parameters of select precursors are found in Supplementary Table 1. The polymerizing components of the resins are described in Supplementary Table 2. It should be noted there exists a lower limit in the amount of crosslinker that can be used to promote 3D PIPS that is determined by practical printing requirements. We found that the printability of the objects begin to degrade substantially when resins contain less than 15 – 20 wt % crosslinker due to insufficient strength of the polymer to support the layer by layer growth of the printed object.

Supplementary Table 1. Resin components and their solubility parameters.

Structure	Name	Molecular weight (g/mol)	Solubility parameter $(MJ/m^3)^{1/2}$
**Polyethylene glycol diacrylate crosslinkers**			
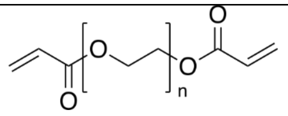	DA-170	170	19.30
	DA-250	250	19.16
	DA-575	575	18.89
	DA-700	700	18.77
**Monomer**			
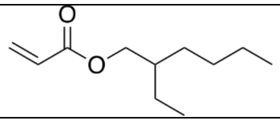	2-Ethylhexyl acrylate (EHA)	184.28	16.90
**Photoinitiator**			
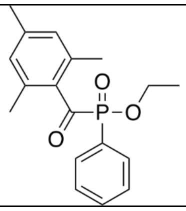	Ethyl (2,4,6-trimethylbenzoyl) Phenylphosphinate (TPO-L)	316.34	
**Non-Polymerizing Component**			
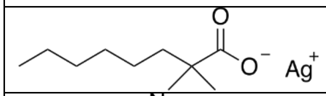	Silver Neodecanoate (AgND)	279.13	20.30*
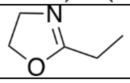	2-Ethyl-2-Oxazoline (EtOx)	99.13	18.71

*Silver neodecanoate solubility parameter was estimated and calculated as neodecanoic acid

Supplementary Table 2. Composition of polymerizing components of photoresins.

Crosslinking Density wt %	Monomer Ethylhexyl Acrylate (g)	Crosslinker DA* (g)	TPO-L Photoinitiator (g)	Total DA-resin* (g)
15	8.4	1.5	0.1	10
20	7.9	2.0	0.1	10
25	7.4	2.5	0.1	10
35	6.4	3.5	0.1	10
50	4.9	5.0	0.1	10
60	3.9	6.0	0.1	10
65	3.4	6.5	0.1	10
80	1.9	8.0	0.1	10
99	0	9.9	0.1	10

*DA-resins DA-170 to DA-700 were prepared using crosslinkers DA-170 (EGDA), DA-250 (PEGDA250), DA-575 (PEGDA575) and DA-700 (PEGDA700).

Supplementary Note 2. Silver content and spatial distribution of silver

The nominal amount of silver present in the 3D printed cylinders is described in Supplementary Table 3 in terms of the weight fraction of silver neodecanoate in the *formulation*, the weight fraction of silver in the *formulation* and the amount of silver in the *sintered sample*, assuming 2-ethyl-2-oxazoline and the neodecanoate is given off during sintering. The amount of silver in the printed object in comparison to the calculated amount of silver in the formulation was confirmed using thermogravimetric analysis (TGA) of cylinders containing 25 wt % AgND (or nominally 9.5 wt % silver post-sintering), as shown in Supplementary Figure 2a and b. The residual amount of silver in the control (no silver) and in a sintered cylinder is -0.03 and 9.66 wt %, respectively, in agreement with the nominal amount of silver. The amount of silver neodecanoate used throughout the manuscript was 25 wt % AgND as highlighted in bold in Supplementary Table 3.

The amount of silver loading was optimized based on the resistance of the cylinders. As shown in Supplementary Table 3, measurable electrical conductivity was observed for resins containing >19 wt % AgND. The resistance of the surface of the cylinders is at a minimum and does not change significantly when the concentration of AgND in the resin was between 25 and 38 wt % while AgND amounts greater than 38 wt % reduced the printing ability and strength of the objects. Optical microscope images of the cylinders printed using 25, 30 and 38 wt % AgND are shown in Supplementary Figure 1.

Supplementary Table 3. Resistance as a function of different wt % of silver precursor (AgND, silver neodecanoate and 2-ethyl-2-oxazoline) in 35 wt % crosslinker DA-250 formulations and their sintered samples.

wt % AgND**** precursor in formulation*	wt % Ag in formulation**	wt % Ag in sintered sample***	Resistance per cm (Ω)	Sheet resistance Ω/□
6.25	1.97	2.38	Not conducting	-
12.50	3.94	4.75	Not conducting	-
18.75	5.91	7.16	20.0 ± 5	6.3
**25.00**	**7.88**	**9.50**	**1.3 ± 0.1**	**0.4**
30.11	9.49	11.88	0.9 ± 0.1	0.3
37.50	11.82	14.25	3.6 ± 0.7	1.1
50.00	15.76	19.00	Poor electrical and mechanical properties	-

*pre-sintering silver neodecanoate content (column 1):

$$wt\ \%\ AgND = wt\ AgND/[wt\ AgND + wt\ resin]\ x\ 100\%$$

**pre-sintering silver content (column 2):

$$wt\ \%\ Ag = wt\ Ag/[wt\ AgND + wt\ resin]\ x\ 100\%$$

***post-sintering silver content (column 3):

$$wt\ \%\ Ag = wt\ Ag/[wt\ Ag + wt\ resin]\ x\ 100\%$$

****AgND defined as silver neodecanoate with 2-ethyl-2-oxazoline in weight ratios 1:0.22.

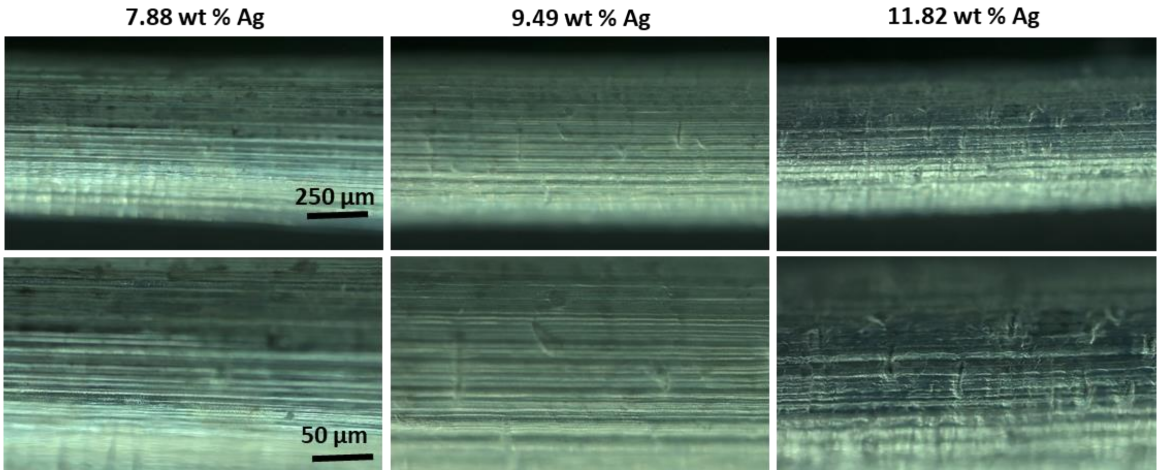

Supplementary Figure 1. Optical microscope images of cylinder printed using 25, 30 and 38 wt % AgND and using resins containing 35 wt % crosslinker DA-250.

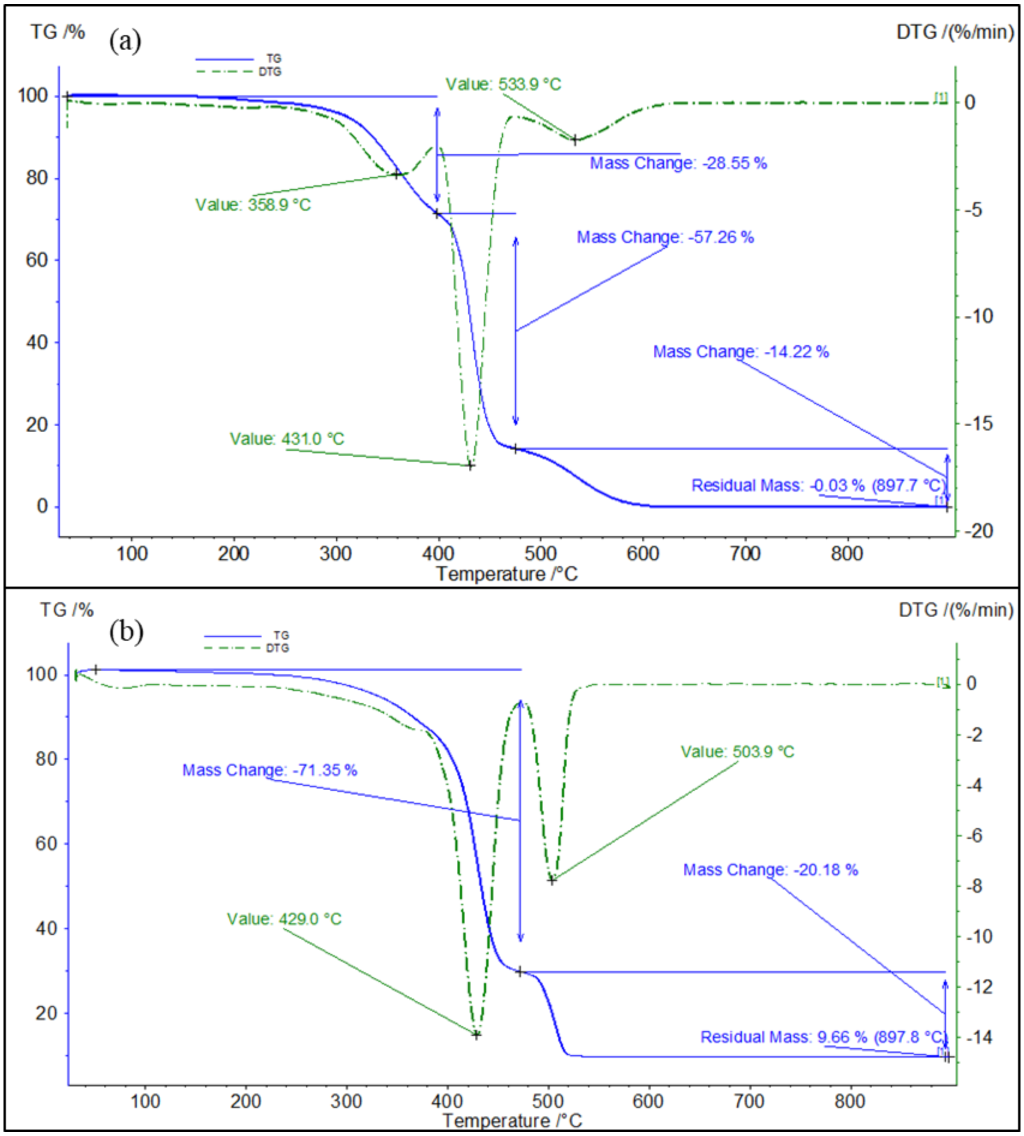

Supplementary Figure 2. TGA of 3D printed and sintered cylinders in air. Cylinders are printed (a) without and (b) with 25 wt % of AgND in the resin.

To verify that silver migrates during the printing process, a formulation known to generate a coating morphology was 3D printed into a cylinder and UV sintered immediately after printing. UV sintering has the effect of converting the silver salt to silver nanoparticles and, thus, by applying UV light to the cylinder, the silver is effectively trapped in space. As shown in the SEM images of Figure 2a in the manuscript, the bright areas at the surface of the cylinders indicate that silver is present at the surface. In a similar experiment, cylinders were printed and left over several days, allowing 2-ethyl-2-oxazoline to evaporate and allowing SEM imaging under reduced pressures. As shown in Supplementary Figure 3, SEM and EDS analysis confirm that the silver is concentrated at the surface of the cylinders.

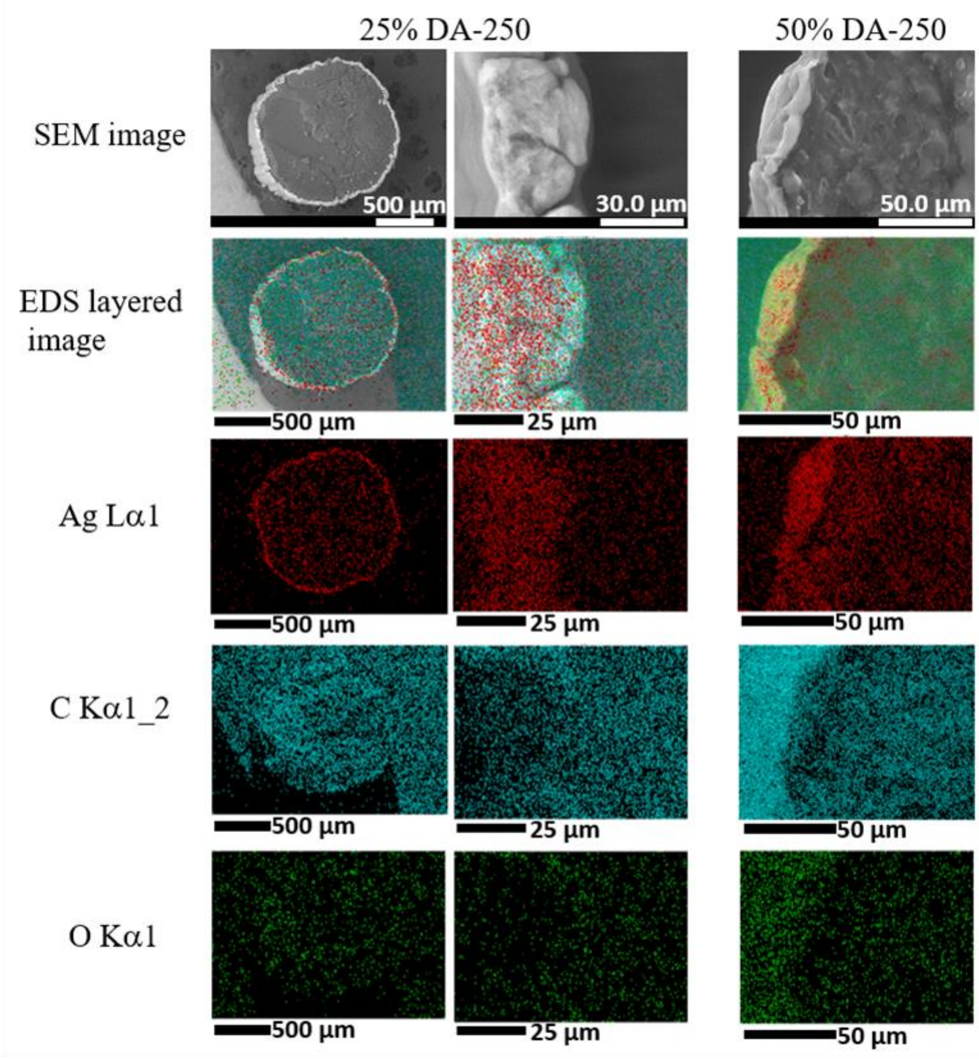

Supplementary Figure 3. SEM images, EDS layered electron images and EDS mapping of silver, carbon and oxygen elements of the cross-sections of pre-sintered cylinders made from two different wt % DA-250 resin compositions. Cylinders were dried for two days in fume hood at room temperature.

The spatial variation of silver varies according to the amount of crosslinked used in the resin. As demonstrated in Supplementary Figure 4a, showing the amount of Ag as a function of distance from the surface of the cylinder as determined by EDS analysis, the higher the crosslinker concentration, the higher the amount of silver trapped within the core of the cylinder. The Supplementary Figure 4b shows evidence of some of the silver inclusion left behind due to silver trapping.

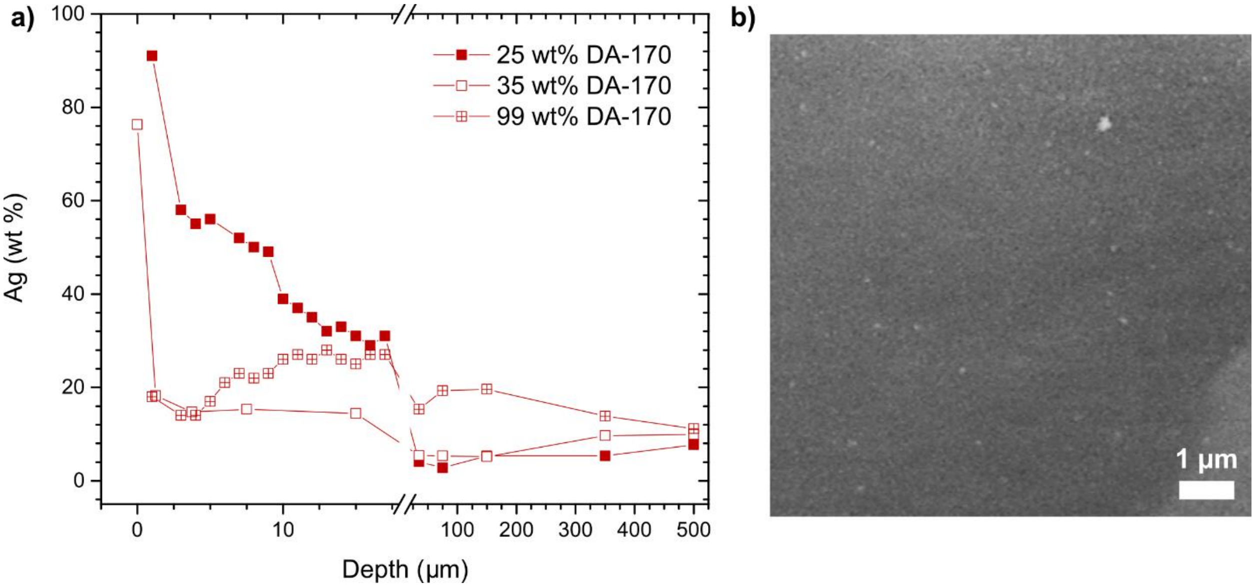

Supplementary Figure 4. a) The Ag wt % as a function of distance away from the surface along the cross-section of a 1.5 mm diameter cylinder. The center of the cylinders made with 25 wt % crosslinkers have as low as 4 wt % Ag while those made with 99% crosslinker have silver concentrations with 15-20 wt % within its core. b) SEM image of the center of 99 wt % DA-575 showing inclusions of silver.

A comparison of antennas made using the phase separation and electroless plating methods is found in Supplementary Figure 5. Optical profilometry measurements (CT-100 by Cyber Technologies) were performed on the surface of each sample to determine relative roughness of each method. The root-mean-square surface roughness (Rq) values of 0.24 and 4.53 µm were found for the antennas prepared by phase separation and electroless-plating of silver, respectively. Photographs of the antennas before and after an adhesion tape test show that the antennas prepared by phase separating silver forms a silver coating with significantly improved adhesion than that of the silver coating generated by electroless plating.

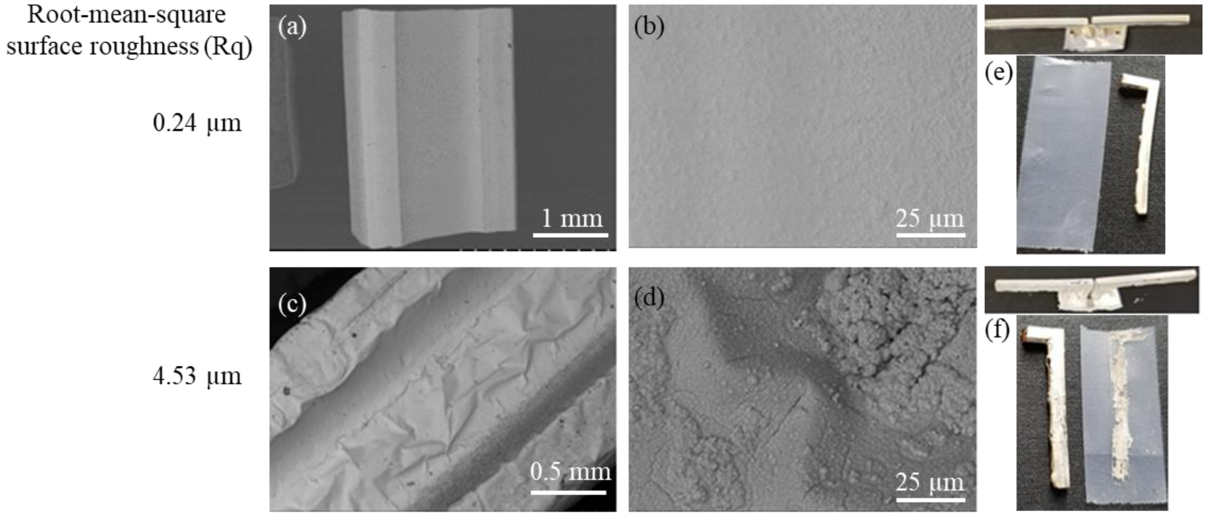

Supplementary Figure 5. SEM images of the surface of dipole antenna prepared using (a, b) phase-separated and (c, d) electroless plating method. The images show the higher surface roughness of the electroless-plated sample compared to the phase separated samples. Roughness values found using optical profilometry show the phase separated samples are one order of magnitude lower than that of electroless deposited samples. Optical microscope images of the samples after a tape adhesion test showing the improved adhesion of the silver coating on samples prepared using phase separation (e) over electroless plating (f).

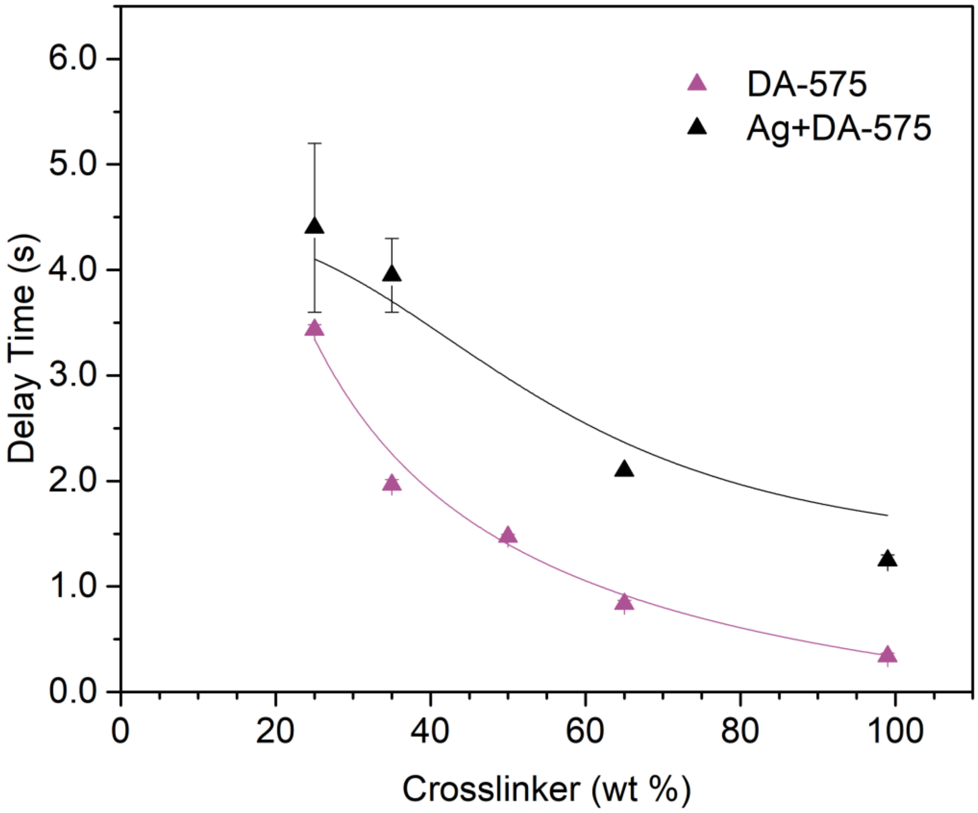

Supplementary Figure 6. Delay time of DA-575 resin with and without Ag precursor as a function of wt % of crosslinker measured using the optical microscope setup. We note that experiments performed using photoresins with AgND show similar trends in t_d as a function of wt % of crosslinker compared to those performed without AgND.

The solubility parameters (δ) of monomers, crosslinkers and the silver complex were estimated using group contribution methods.[2] From these values, the solubility parameters of the monomer/crosslinker mixtures of each resins were estimated by calculating a volume weighted solubility parameters. These values are plotted in Supplementary Figure 7 and compared to the δ value of the 2-ethyl-2-oxazoline and neodecanoic acid (purple and blue horizontal line, respectively).

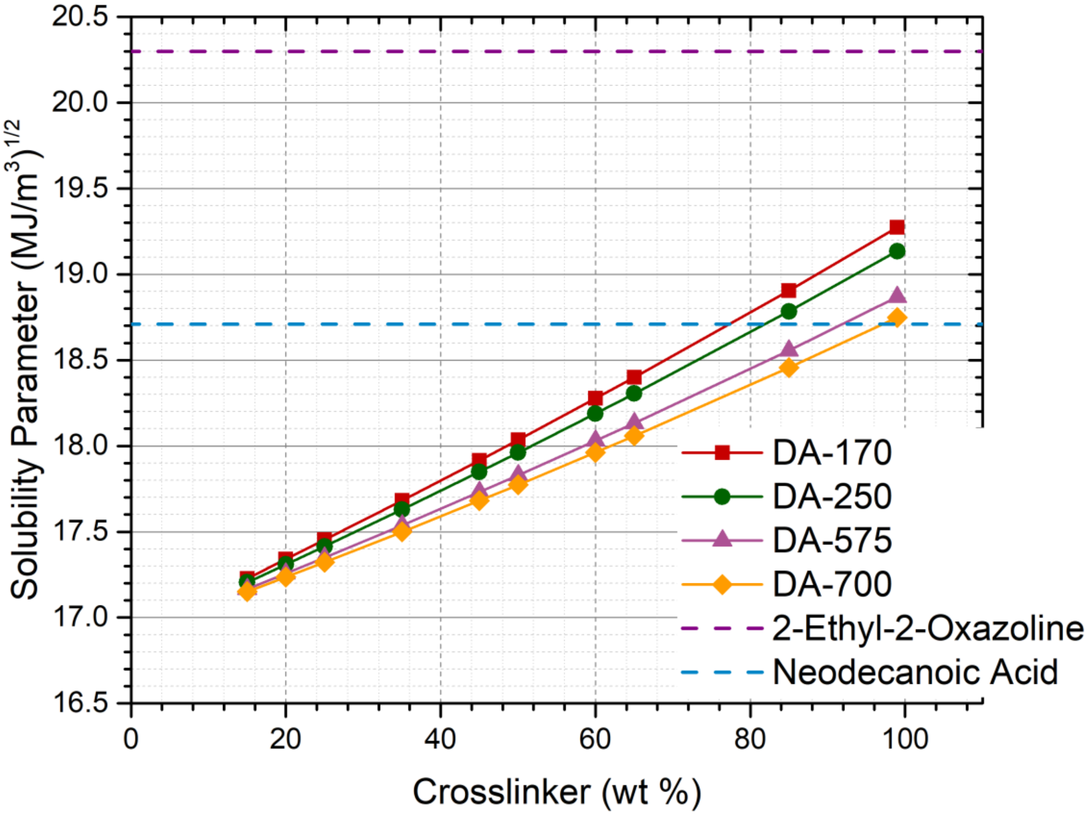

Supplementary Figure 7. Solubility parameters of monomers, crosslinkers and the silver complex estimated using group contribution methods.

Supplementary Note 3. Trusses, antennas and anti-microbial structures

Piezoresistive truss sensors

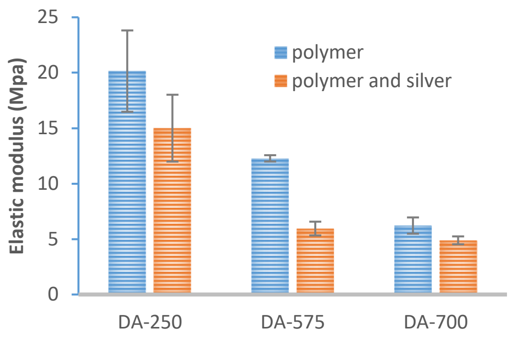

Supplementary Figure 8. The elastic modulus of the various polymers using 25 wt % crosslinker. With increasing crosslinker segment, the elastic modulus decreases. Elastic modulus was determined from tensile tests on samples approximately 1.5 mm in diameter and 1 cm in length. The test instrument, a LEX820/FDAS770 automated fiber tensile test system from Dia-Stron (Andover, UK), measured the sample cross-section using an on-board laser micrometer followed by tensile testing using a high resolution extensometer with 20 N load cell. Four samples of each type were measured to report the average result and standard deviation.

The trusses, with the design shown in Supplementary Figure 9a and dimensions of 11.24 x 11.24 x 13.40 mm, were 3D printed using resins containing various crosslinker concentrations. Compression cycling of the truss structures showed trusses made with 39, 46, 52 and 58 wt % crosslinker responded to a 250 μm compression with maximum changes in resistance of 2.5, 6.0, 12.5, and 48 % respectively.

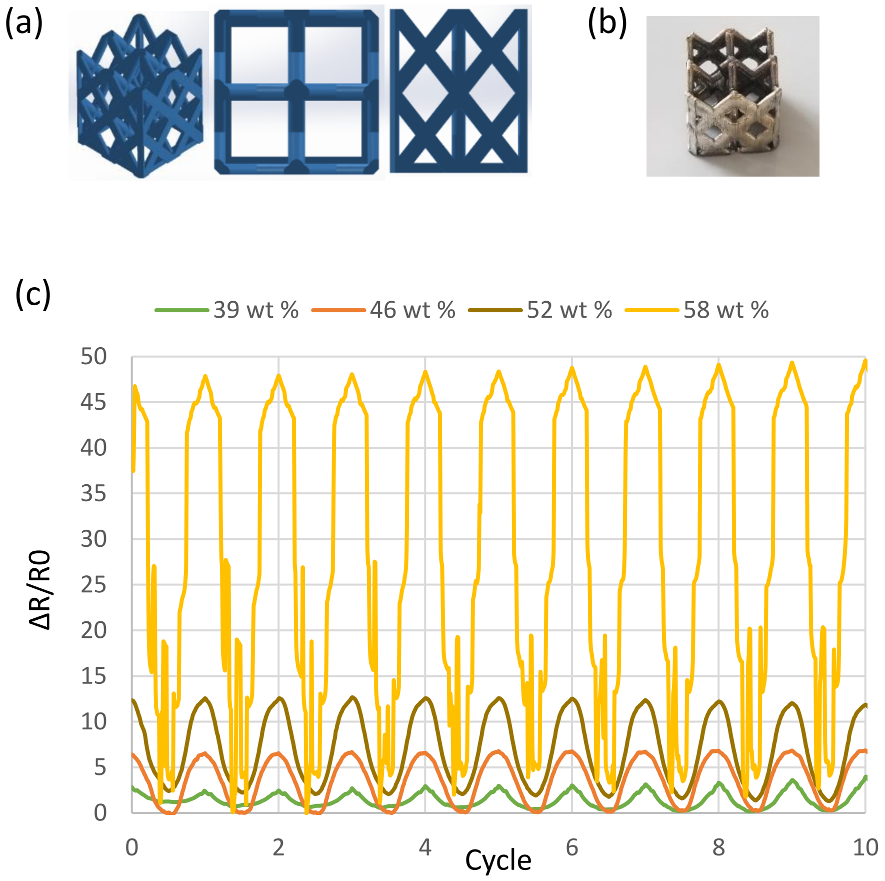

Supplementary Figure 9. a) Isometric, top and side views of the truss structure used in the strain sensor experiments. b) Photograph of 3D printed truss with dimensions of 11.24 x 11.24 x 13.40 mm. c) Relative resistance of trusses under compression for trusses made with resins containing various crosslinking concentrations.

.

Antennas

The measurement of gain was performed in an anechoic chamber using a gain standard horn antenna as shown in Supplementary Figure 10a below. The gain standard was used as the receive antenna (antenna array in Figure 6) and s-parameter measurements collected as they were for the antenna array with the VNA. With a peak measured s-parameter magnitude of -54.8 dB and a known maximum gain for the standard of 7.8 dB gives a conversion of

$$G = S_{21} + 62.6\ dB \tag{1}$$

where G is gain and S_{21} is the magnitude of transmission, both in dB. This equation allows the s-parameter data to be converted to gain values assuming negligible return loss. In this case both the gain standard and the printed dipole array were matched to 50 Ω at 2.4 GHz with return loss values better than -10 dB.

Making the s-parameter measurements of the antenna array and converting to gain using Equation 1 gives the plot as shown in Supplementary Figure 10b. This measured data is shown plotted beside simulated data from Ansys HFSS.

In HFSS, the array model (shown in Supplementary Figure 10c) used a finite surface impedance for all conductors of $1 \times 10^6\ S/m$. From Supplementary Figure 10b, the simulated results show 4.1 dB more gain than the measured results. This discrepancy in results could be due to losses from the conductive adhesive used to join the dipole antenna branches to the conductor or the PCB header pins used as through hole vias to connect the dipoles through to the ground plane. It could also be due to a breakage that happened in the middle of one antenna during measurement. The break was repaired with copper tape and checked for DC conductivity, but its gain could have been reduced by the repair. This could also explain the Asymmetry in the measured radiation pattern with a significant side lobe at an angle of $\theta = 90°$.

Other dipole antennas have been 3D printed such as shown in literature where the design was metalized with copper air brushing and set a quarter wavelength above a ground plane for enhanced radiation.[3] The maximum reported gain for the dipole in literature was 6.5 dB.[3] To close the gap between this number and the 1.3 dB reported here, the dipoles could be optimized for interaction with a ground plane by elevation to a quarter wavelength for enhanced gain from the ground plane reflection. Thicker vias could create less inductance for a stronger connection between the ground plane and the base of the dipole. A high conductivity adhesive such as copper tape could be used instead of conductive epoxy to reduce transition losses at the antenna. Adding a balun at the input to each dipole could also reduce transition losses and improve the overall gain of the array.

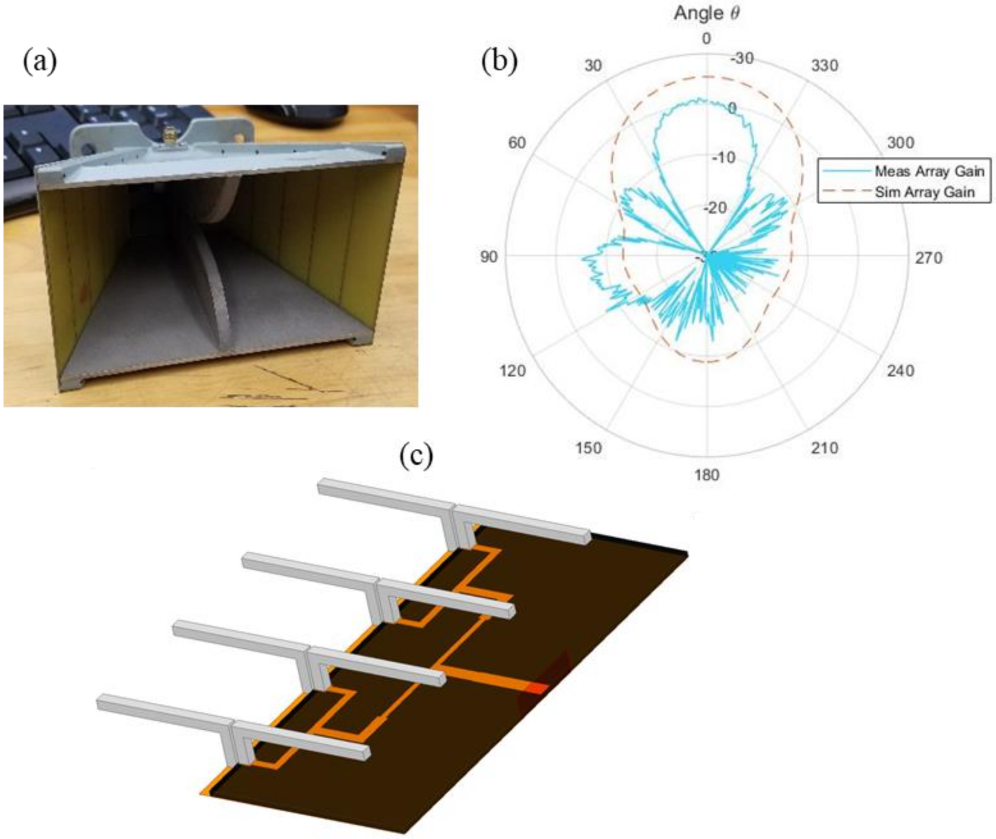

Supplementary Figure 10. (a) Gain standard horn antenna (BAE Systems H-1498). (b) Measured antenna array gain compared with HFSS simulation. (c) HFSS model of dipole antenna array with feeding network and metal conductivity of 1×10^6 Siemens/m.

Anti-microbial objects

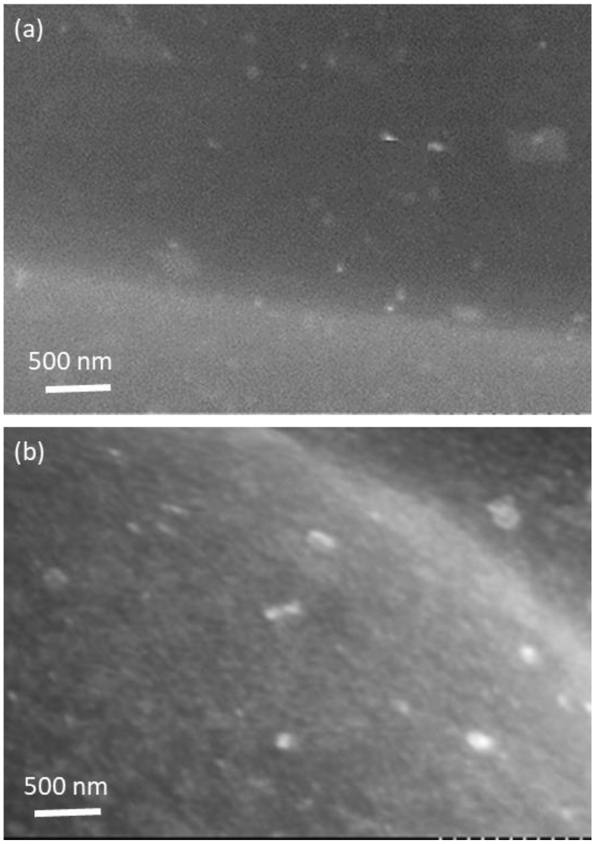

Supplementary Figure 11. An SEM image of the surface of 3D printed the anti-microbial sample made from resins containing a) 0.5 wt % and b) 1.0 wt % AgND.

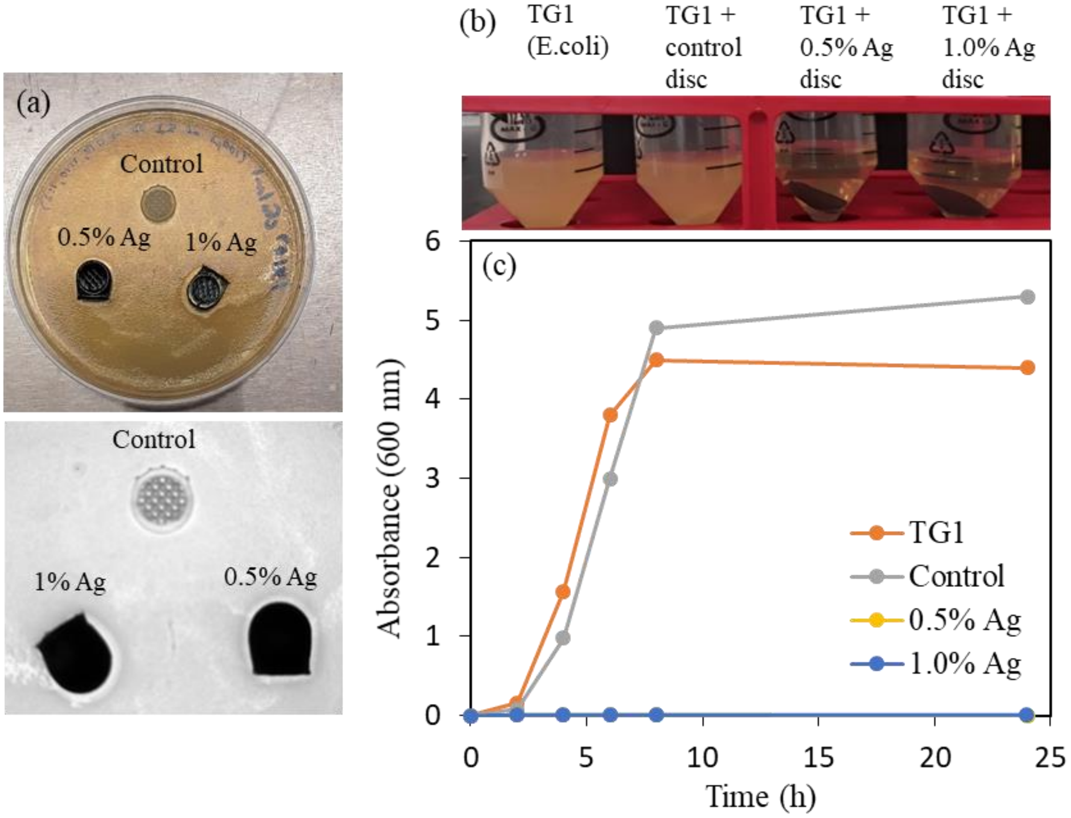

Supplementary Figure 12. (a) Photographs of the bacterial inhibition zones created by the different scaffolds against TG1 (E.coli) and (b, c) bacterial growth kinetics in liquid medium of E.coli.

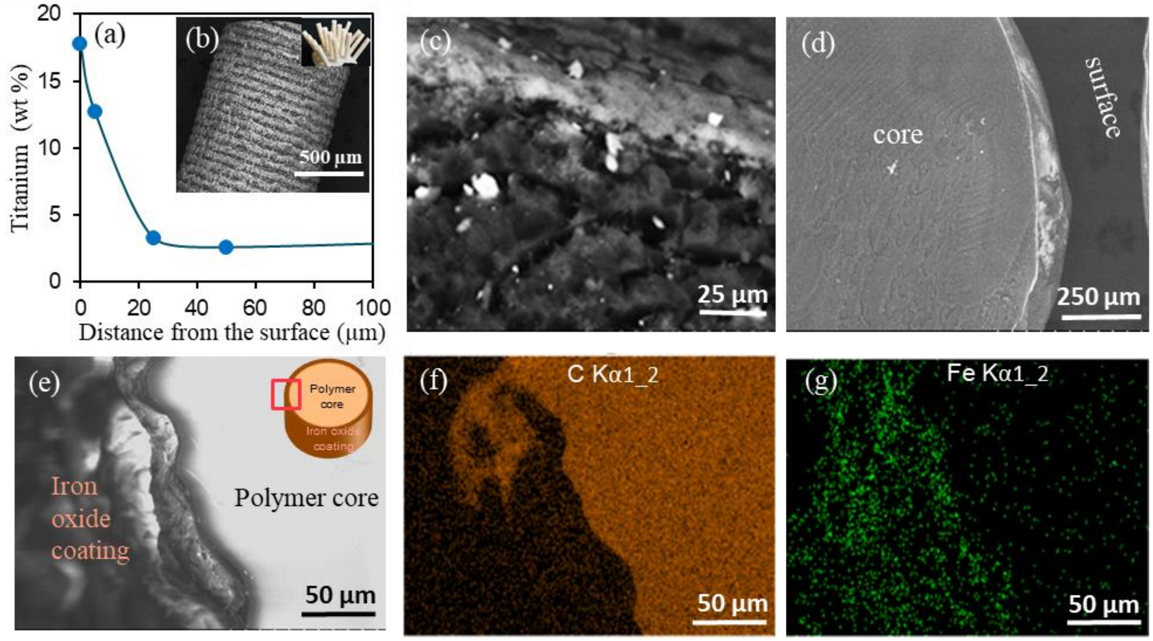

Supplementary Figure 13. (a) Variation of the titanium as a function of distance from the surface of a cylinder printed with TiO_2 nanoparticles and as measured by EDS. (b) SEM image of surface of cylinder printed with TiO_2 nanoparticles and image of printed cylinders (insert). (c) Cross-sectional SEM image of a cylinder printed with TiO_2 nanoparticles. (d) Cross-sectional SEM image of a cylinder printed with barium strontium titanate nanoparticles. (e – f) SEM/EDS cross-section image of a cylinder printed with iron oxide nanoparticles. The nanoparticles appear as bright areas in the SEM. EDS mapping of carbon (f) and iron (g) elements in the sample.

Supplementary Note 4. Light intensities and delay times

Different light intensities for 3D printing were explored in order to establish optimum printing conditions. The light intensities tested were 350, 560 and 760 W/cm^2 as shown in Supplementary Table 4. The resistances of the cylinders printed under these light intensities are shown in Supplementary Figure 14 and show no significant differences. These results suggest that light intensity does not affect the phase separation of silver significantly within the range of 350 to 760 W/cm^2.

The gelation experiments that were used to acquire delay times employed a light intensity of 566 W/cm^2 and falls within the range of light intensities used for printing. As the resistance did not vary significantly within this range, the gelation behavior of the resins is expected to be the same for printing and gelation experiments, thus, validating our use of delay times as a relative measure of gelation rates for 3D printed objects. The gelation experiments were done under constant illumination/no moving laser rate so dose couldn't be calculated.

Supplementary Table 4. Light intensity and estimated light doses of the laser used for printing and gelation experiments.

	**Printing (laser setting 58)**	**Printing (laser setting 66)**	**Printing (laser setting 75)**	**Gelation experiment**
Spot radius (μm)	35	35	35	0.75
Spot radius (cm)	0.0035	0.0035	0.0035	0.000075
Spot area (cm^2)	3.85E-05	3.85E-05	3.85E-05	1.77E-08
Laser power (mW)	13.5	21.7	29.2	0.01
Light intensity (W/cm^2)	350	560	760	566
Linear printing rate (cm/s)	8.5	8.5	8.5	N/A
Light dose (mW•s/cm^2)[4]	360	580	780	N/A

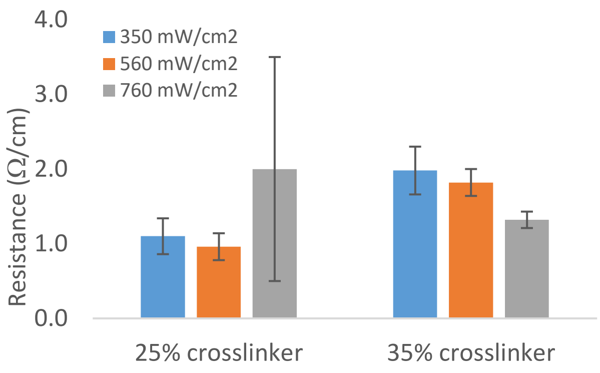

Supplementary Figure 14. Resistance of cylinders printed using different light intensities for DA-250 resins.

The thermal stability of the polymer was tested by isothermally heating the polymer sample at 210°C and monitoring its mass loss by TGA. Supplementary Figure 15 shows insignificant amount of polymer decomposition confirming that the polymer does not degrade during the silver sintering step.

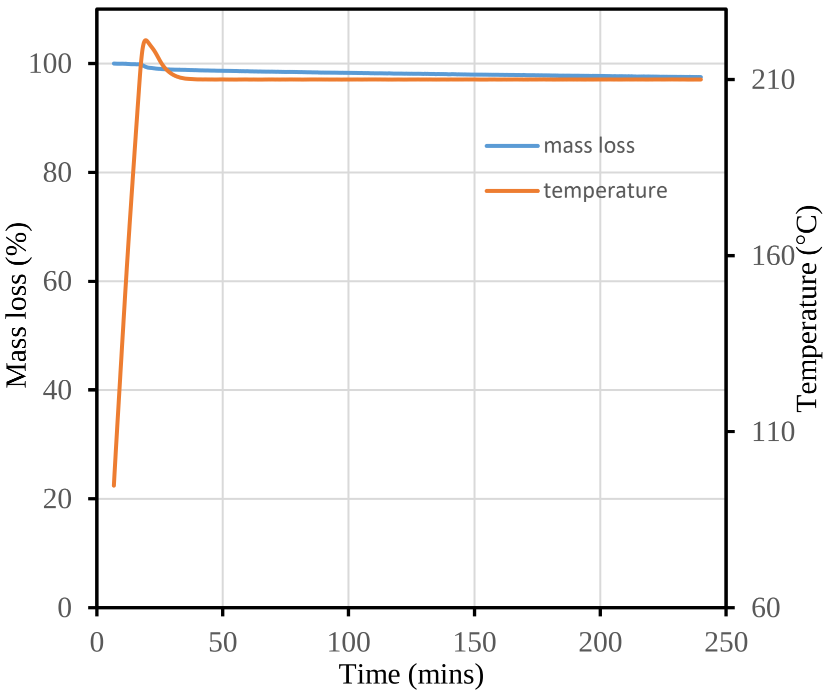

Supplementary Figure 15. TGA of pre-sintered 3D printed cylinders using a resin mixture (75 mL of 50 wt % DA-575 with 25 mL of 35 wt % DA-250) that contained no silver and treated with 5 minutes of UV curing. The mass loss of the cylinders were analyzed by TGA under isothermal condition at 210°C (i.e. sintering temperature). The mass loss after 1 h is 1.4% demonstrating that the polymer does not degrade significantly during the sintering step.

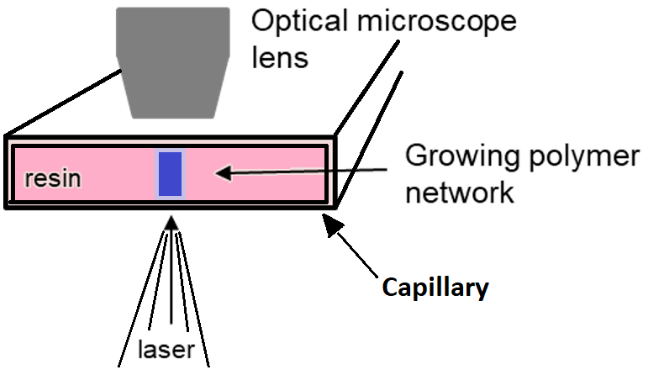

Supplementary Figure 16. Optical microscope setup for examining the formation of the polymer network and measuring delay time upon exposure to a 405 nm laser.

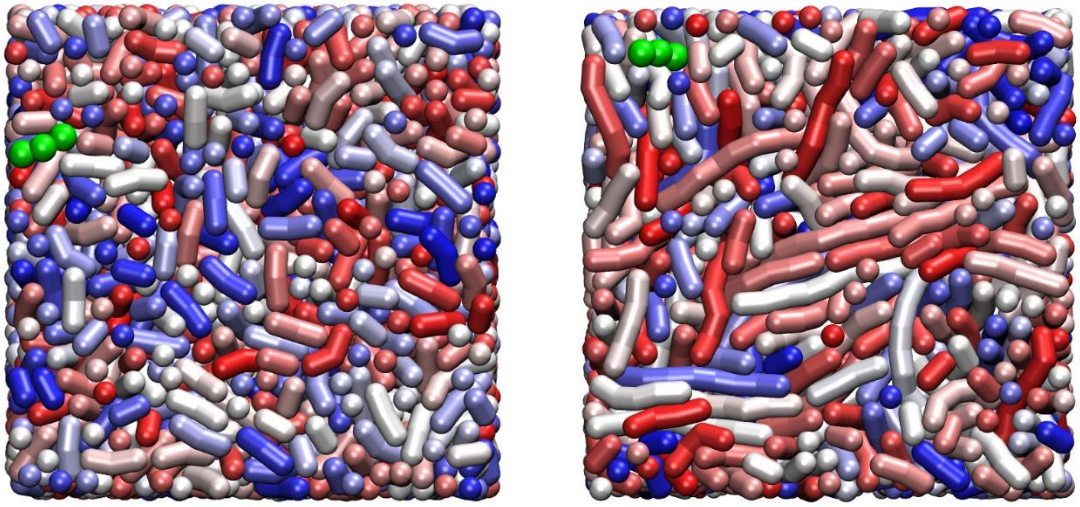

Supplementary Figure 17. Images of the initial state of the system for crosslinkers of length 3 (left) and length 9 (right). The probe molecule is shown in green and is represented as it is modeled: three beads connected by springs. For clarity, the crosslinker molecules are shown as tubes such that individual molecules are easier to distinguish. Note however that these molecules are also modeled as beads connected by springs. These images are cross-sections selected to show the location of the probe molecule. Instances of shorter crosslinker molecules, or even single beads, are an artefact of cross-sectional view of molecules.

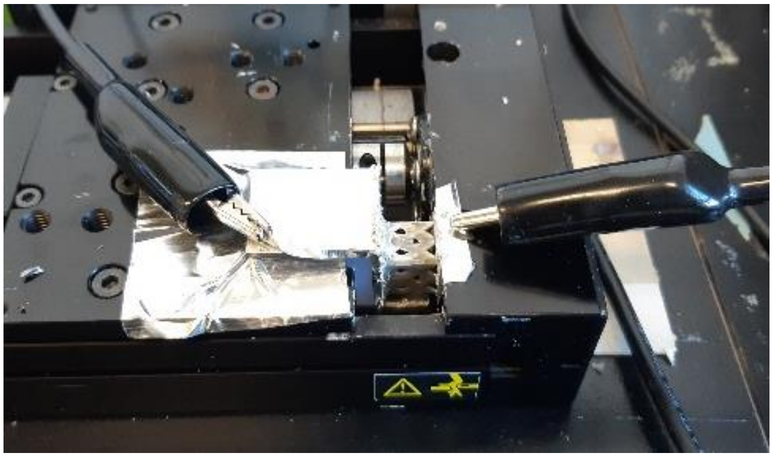

Supplementary Figure 18. Experimental setup for the strain sensor measurement. The sample dimensions are 11.24 x 11.24 x 13.40 mm.

Note for Figures: All our error bars are based on standard deviations.

Supplementary References

1. Titkov, A. I., Logutenko, O. A., Bulina, N. V., Yukhin, Y. M. & Lyakhov, N. Z. Synthesis of nonspherical nanoparticles by reducing silver neodecanoate extract with benzyl alcohol. Theor. Found. Chem. Eng. 51, 557–562 (2017).
2. Van Krevelen, D. W., Krevelen, D. W. & te Nijenhuis, K. Properties of polymers: Their correlation with chemical structure; their numerical estimation and prediction from additive group contributions. (Elsevier, 2009).
3. Ranjbar Naeini, M., Mirmozafari, M. & Van Der Weide, D. Monolithic 3-D Printing of an Integrated Marchand Balun with a Dipole Antenna. IEEE Trans. Components, Packag. Manuf. Technol. 10, 654–658 (2020).
4. Zakeri, S., Vippola, M. & Levänen, E. A comprehensive review of the photopolymerization of ceramic resins used in stereolithography. Addit. Manuf. 35, 101177 (2020).
