# Supplementary material for: Direct printing of functional 3D objects using polymerization-induced phase separation
Source: Nat Commun. 2021 Jan 4;12:55. doi: 10.1038/s41467-020-20256-3 (PMC7782741; doi:10.1038/s41467-020-20256-3)
Supplement: Supplementary file 3 — Description of Additional Supplementary Files [file 41467_2020_20256_MOESM3_ESM.pdf]

Description of Additional Supplementary Files

Supplementary Movie 1: Optical microscope capture of polymer network island growth of 15 wt % DA-170 resin.

Supplementary Movie 2: Optical microscope capture of polymer network island growth of 99 wt % DA-700 resin.

Supplementary Movie 3: Optical microscope capture of polymer network island growth of 99 wt % DA-700 resin with laser on time and sliced area highlighted.
